# Oura Ring Behavioral Feedback Intervention for Alcohol Reduction in Young Adults: User Experience Evaluation of a Pilot Randomized Trial

**DOI:** 10.2196/78613

**Published:** 2025-12-04

**Authors:** Frances J Griffith, Oksana K Ellison, Sahiti Kunchay, Madilyn Augustine, Kelly S DeMartini, Michael Fatigate, Leah Latimer, Stephanie S O'Malley, Nancy S Redeker, Garrett I Ash, Lisa M Fucito

**Affiliations:** 1 Department of Psychiatry, Yale School of Medicine, Yale University, 300 George Street, New Haven, CT, 06511, United States, 1 859 257 6841; 2 Department of Psychology, University of Kentucky, Lexington, KY, United States; 3 School of Nursing, University of Connecticut, Storrs, CT, United States; 4 Department of Biomedical Informatics and Data Science, Department of Internal Medicine, Section of General Internal Medicine, Yale School of Medicine, Yale University, New Haven, CT, United States; 5 Veterans Affairs Connecticut Healthcare System, Pain, Research, Informatics, Medical Comorbidities and Education Center, West Haven, CT, United States

**Keywords:** wearable technology, digital health, mobile health, alcohol use, drinking, heart rate variability, sleep, personalized feedback, young adults, natural language processing, mobile phone

## Abstract

**Background:**

Wearable fitness technologies, like the Oura Ring (Oura Health Oy), provide physiological metrics, like sleep and heart rate data, to a growing user base of young adults. However, these technologies and connected mobile apps do not measure young adults’ alcohol use that contributes to these metrics. Personalized feedback on the impact of alcohol on sleep and heart rate may boost motivation to reduce drinking among young adults.

**Objective:**

For this pilot randomized controlled trial, we evaluated the acceptability, feasibility, and perceived effectiveness of a wearable personalized feedback intervention for alcohol reduction in young adults that integrated physiological and behavioral data.

**Methods:**

Recruitment took place offline and online via open access websites. Participants (N=60) wore the Oura Ring for 6 weeks and completed daily behavioral smartphone diaries. Only the feedback group (n=30) had full access to the Oura Ring app and personalized feedback reports every 2 weeks, received from the study team. The app included daily feedback on sleep and cardiovascular recovery. Feedback reports combined Oura Ring and diary data to show trends of alcohol use alongside sleep and cardiovascular data. We used artificial intelligence–driven convergent mixed methods to evaluate self-assessed exit surveys and face-to-face exit interviews, including natural language processing and researcher-coded qualitative analyses with interviews.

**Results:**

Half of participants (30/60, 50%) were men, 81.6% (49/60) were White, and they had a mean age of 22.02 (SD 1.98) years. Across both groups, the overall program was described as highly acceptable, feasible, and effective. Wearing the Oura Ring was highly acceptable and feasible. The smartphone diaries were moderately acceptable, moderately-highly feasible, and highly effective. The feedback reports were highly acceptable, feasible, and effective. Among feedback group participants, the Oura Ring and app were moderately effective. The feedback group participants also had high adherence using the app daily, and 80% (48/60) read all 3 feedback reports. Per natural language processing, the most common topic in the feedback interviews related to their behavior change due to multiple intervention components (*Ɵ_k_*=0.18). This contrasted with the most common topic from assessment group participants about prechange learning (*Ɵ_k_*=0.22). During the researcher-coded qualitative analysis, we identified themes in 3 categories. Most participants described helpful aspects of the Oura Ring and app, smartphone diaries, and feedback report. Most felt that the report had the right amount of information, and a large group reported they learned about their sleep deficits. Curiosity was the most common reason participants joined the study. SMS text messages and usability kept them engaged, and almost none considered dropping out.

**Conclusions:**

Commercial fitness wearables that integrate behavioral data may be acceptable and feasible and promote readiness to change drinking in young adults who are generally unconcerned about risky behaviors.

## Introduction

### Physiological Feedback for Alcohol Use in Young Adults

Wearable fitness technologies (eg, smartwatches and smart rings) are increasingly popular among young adults but may be missing crucial behavioral health data to guide behavior change. Over half (52%) of young adult consumers in the United States own commercial wearables and use them for fitness, stress or weight management, sleep, and other wellness goals [1]. Their appeal lies in their ability to reliably measure physiological signals (eg, heart rate, blood oxygen, and skin temperature) in a noninvasive, accessible way via biometric sensors [2]. However, physiological data are provided to users without information about concurrent behaviors that impact physiological states and patterns [3], including alcohol use. This leaves users without guidance for how to change risky behaviors to support their wellness goals.

Alcohol use disorder (AUD) onset and rates of heavy drinking peak during young adulthood, but young adults are often more concerned about their general wellness than alcohol use behaviors [4-7]. However, risky alcohol use can impede wellness goals, like sleep improvement and cardiovascular recovery [8,9]. Alcohol’s harmful effects across the body are well-established and include effects on cardiovascular health, sleep, and immune function [10]. Alcohol use can contribute to cardiac arrhythmias [8] and poor sleep quality in young adults, and vice versa [9,11,12]. Inadequate sleep may lead to increased, problematic alcohol use in young adults [12-15] and to relapse for people with AUDs [16,17]. Furthermore, behavioral interventions for insomnia may reduce alcohol use in adults who drink heavily [15], especially digital insomnia programs [18] and broader digital sleep interventions [5]. Therefore, wearable fitness technologies may support sleep and other wellness goals by targeting related risky behaviors, like alcohol use.

Personalized feedback from wearable technologies may help young adults make connections between their alcohol use and their wellness goals, like improved sleep [19]. In reviews and meta-analyses of clinical trials [20,21], digital personalized feedback interventions result in small but meaningful reductions in young adults’ drinking. Feedback interventions often involve normalized feedback comparing young adults’ perceptions of peer drinking and actual peer drinking levels, which highlights that young adults’ peers often drink less than they expect [20]. This feedback then facilitates comparison of their own self-reported drinking to actual norms. Personalized feedback for alcohol reduction is also increasingly integrating multiple personal data streams, enabling comparison between drinking and other facets of young adults’ experiences [21]. Combining physiological data (eg, alcohol’s effects on heart rate and sleep) and self-reported behavioral data (eg, number of drinks consumed) can provide highly personalized feedback and insights that can increase user engagement in interventions [3], which is critical to intervention effectiveness [22].

Feedback in digital health interventions tends to be delivered (1) when a behavior is occurring or (2) after a behavior occurs [23], which facilitates reflection-in-action or reflection-on-action, respectively, to promote insight [24]. Retrospective delivery of feedback allows users to think about their behavior in the larger context of related events, feelings, and motivations and to consider relations between their experiences and physiological or behavioral data [25]. Feedback with a record of experiences over time can facilitate “descriptive reflection” (revisiting behaviors) and “explanatory reflection” (explaining behaviors). Furthermore, feedback showing correlations or causal patterns (eg, associations between alcohol use and poor sleep) can encourage “dialogic reflection” [25]. Reflecting on causal patterns between alcohol use and hindered wellness goals may boost motivation to change alcohol consumption among young adults who are unconcerned about their alcohol use [5].

Popular commercial devices are uniquely positioned to promote reflection and insight among young adults who might not otherwise seek help with risky behaviors, like alcohol use [25]. Given the potential for wearable technologies to promote wellness and decrease risky behaviors simultaneously [5], it is essential to study young adults’ experiences with integrated physiological and behavioral feedback on alcohol use. User engagement is critical to the success of digital health tools [22], and intervention designers must understand how to optimize feedback to encourage long-term engagement from young adults.

### This Study

The current study is the first randomized controlled trial (RCT) of a wearable, personalized feedback intervention for young adults with risky drinking that combines: (1) physiological metrics of sleep, heart rate variability (HRV), and resting heart rate via wearable photoplethysmography biosensors in the Oura Ring (Oura Health Oy) and (2) behavioral daily diary self-monitoring of sleep and alcohol use.

Our primary evaluation aim was to describe young adults’ perceptions of the acceptability, feasibility, and perceived effectiveness of the Oura Ring wearable, the Oura Ring mobile app, smartphone diary self-monitoring, and personalized feedback and tailored advice reports, with a focus on participants’ experiences in the feedback group. In addition, we also had some exploratory aims. First, we aimed to compare user experiences between the feedback group (full access to the Oura Ring mobile app and feedback reports every 2 weeks) and the assessment group. Second, we aimed to compare user experiences of different intervention components and, finally, explore young adults’ health coaching preferences for personalized feedback.

## Methods

### Study Design

For this pilot, parallel-group RCT, our goal was to evaluate users’ experiences with a wearable, personalized feedback intervention leveraging physiological data (cardiovascular and sleep) and behavioral data for alcohol reduction. Participants were randomly assigned 1:1 to either the feedback (n=30) or assessment (n=30) group. In 2022, all participants wore the commercial wearable Oura Ring, Gen2 (Oura Health Oy) daily for 6 weeks, completed daily smartphone diaries about their sleep, alcohol or substance use, and health behaviors, and completed follow-ups at weeks 6 and 10. Study staff members gave SMS text reminders to participants to sync Oura Ring data 3‐4 times per week and to complete smartphone diaries (if not yet completed).

The feedback group had full access to the Oura Ring mobile app via smartphone. The app included daily personalized, biometric feedback on sleep (ie, sleep stages, wakefulness, timing, efficiency, latency, and duration), physical activity (ie, calories burned and steps), cardiovascular recovery (HRV and resting heart rate), respiratory rate, and body temperature. Furthermore, the app provided composite health scores in the areas of “sleep,” “activity,” and “readiness” based on proprietary algorithms and in-app sleep tips and activity prompts. The assessment group only had partial access to the Oura Ring mobile app, including general wellness tips, but they did not have access to personalized, biometric feedback in the app (eg, daily sleep and cardiovascular feedback). Based on our previous work [4-7], we judged this to be the best control as it provides the experience of wearing the ring, having knowledge of being monitored, and using the app.

The feedback group also received personalized feedback and tailored advice reports every 2 weeks, derived separately and delivered by our research team, on integrated physiological Oura Ring data and behavioral smartphone diary data. Personalized feedback included trends of alcohol and other substance use based on self-report (eg, heavy drinking or substance use occasions, drinks per week, and peak blood alcohol level), alongside cardiovascular recovery and sleep data over each 2-week period (refer to the studies by Fucito et al [4,7] for more details on similar feedback reports in previous research). Reports included data visualization to reveal patterns among sleep, cardiovascular, and alcohol and substance use data streams. Within reports, participants were also given brief, evidence-based advice tailored to their data, such as sleep hygiene, controlled drinking, stress management, and exercise strategies. The assessment group did not receive feedback reports every 2 weeks from the research team, but received 1 delayed feedback report postintervention at week 10. The assessment group participants were unblinded given their knowledge that features of the Oura Ring app were blocked and that they were not receiving feedback reports throughout the intervention period. Furthermore, study team members were unblinded when administering participant appointments and interviews.

### Recruitment

Participants were recruited through online advertisements on open-access social media sites (eg, Snapchat [Snap Inc], Instagram [Meta], Facebook [Meta], and Reddit) and offline via community flyers in universities, gyms, and other public spaces. Although advertisements did not explicitly seek out young adults who wanted to reduce their drinking, they did target young adults with heavier drinking levels as central to the study. Interested volunteers were directed to complete an online screener. The study’s affiliation with Yale School of Medicine was apparent in recruitment and screening materials. To be eligible, participants needed to (1) be 18‐25 years old, (2) be fluent in English, (3) report ≥4 heavy drinking occasions (≥5 drinks for men and ≥4 for women) in the past 28 days, (4) be at risk of harmful drinking (Alcohol Use Disorder Identification Test- Consumption [26] ≥7 for men or ≥5 for women), and (5) own a smartphone. Potential participants were excluded if they had (1) current (active) alcohol or sleep treatment; (2) clinically severe AUD withdrawal or substance use disorder other than cannabis in the last 12 months as assessed by diagnostic interview; (3) severe symptoms of a mental health disorder (MHD), for example, current psychosis or suicidality; (4) history of sleep disorders; (5) job with a night or rotating shift that prevented a consistent sleep schedule; or (6) travel >2 time zones during study participation or a month before.

Of the 81 participants who were screened online for eligibility, 21 did not meet the inclusion criteria due to insufficient drinking levels (n=15), severe MHDs (n=2), and planned travel (n=1; Multimedia Appendix 1). Furthermore, 3 participants were no longer interested. Those 60 participants who met online screening eligibility were invited to attend an intake to confirm eligibility face-to-face. All 60 eligible participants who were enrolled and randomized into groups ultimately completed the treatment, and 59 completed follow-up. Given that this was a pilot trial, neither a power analysis nor other sample size calculations were undertaken. We judged that 60 participants, including 30 in the feedback group, would be sufficient to assess intervention feasibility.

### Evaluation Procedure

To assess user experiences, participants completed self-assessed, web-based exit surveys and face-to-face exit interviews designed for this study (Multimedia Appendix 2). Acceptability was defined as survey ratings of intervention satisfaction and likeability and interview sentiment and descriptions of preference. Feasibility was determined via survey ratings of intervention comfort, schedule workability, life interference, and interview descriptions of understandability. Perceived effectiveness encompassed survey ratings of intervention helpfulness alongside interview descriptions of helpfulness, behavior influence, and behavior change. The timing and some content of assessments varied by group. To assure quality, exit interviews were given face-to-face, and exit surveys were self-assessed by participants under the supervision of a study team member.

### Exit Surveys

All participants completed exit surveys at week 6. Participants were emailed a web link to the exit survey, which they could complete via smartphone or computer. Exit surveys included original Likert scale and Likert-type questions written by the study team regarding the acceptability, feasibility, and perceived effectiveness of the overall program, wearing the Oura Ring, and completing the smartphone diaries. These user experience questions were based on validated measures [27,28] and have been used in a variety of previous online user experience studies [5,29,30]. In the week 6 exit survey, the feedback group participants responded to additional questions about the acceptability, feasibility, and perceived effectiveness of the Oura Ring mobile app and the personalized feedback and tailored advice reports received every 2 weeks. Questions about the perceived effectiveness of the intervention referred to the helpfulness of information and tips for reducing alcohol and improving sleep or cardiovascular health. At week 10, the assessment group participants were not asked questions about the Oura Ring mobile app due to their limited access, but they were asked questions about the delayed feedback report that they received. After trial initiation, additional survey questions were completed by a smaller subset of participants, including their willingness to purchase the Oura Ring (n=51) and feedback reports (n=53), the likelihood of recommending the Oura Ring to others (n=51), the likability and understandability of alcohol or substance information in feedback reports (n=32), and the helpfulness of behavioral tips (such as sleep and alcohol tips) in feedback reports (n=28‐31).

### Exit Interviews

Both groups also completed exit interviews with a study team member (MA). The feedback group participants completed exit interviews during week 6 follow-ups. Interview questions focused on changes in overall health, sleep, and alcohol or substance use during the study, comparisons to peers, helpfulness of received intervention components (Oura Ring, smartphone diaries, and personalized feedback reports), interests and preferences for health coaching, and suggestions for future research. The feedback group participants also answered questions about the Oura Ring mobile app and provided comparisons among components. Thematic saturation occurred before interviewing all feedback group participants, but all participants were asked to take part in interviews to ensure all user experiences were captured. Exit interviews were not initially planned for the assessment group but were added after study initiation to their week 10 follow-up to gain a richer understanding of their experiences and reactions to the delayed feedback report. However, this protocol addition resulted in the first assessment group participants (n=4) not being offered interviews. Assessment group participants were not asked questions about the Oura Ring mobile app because they did not have full access, and they were not asked to compare intervention components. Consistent with iterative qualitative research methods [31], some interview questions for both groups (eg, exploratory health coaching questions) were adaptively added during the evaluation process in response to users’ experiences.

### Data Analysis

We used an innovative convergent mixed methods approach [5] incorporating artificial intelligence (AI)–driven natural language processing (NLP) to evaluate this wearable, personalized feedback intervention for young adults with risky drinking. Exit surveys and exit interviews were analyzed in parallel to assess the convergence of findings. For our primary aim, we examined the descriptive results of exit surveys to evaluate the acceptability, feasibility, and perceived effectiveness of the overall program and its intervention components. Then, for our exploratory aims, we assessed (1) predictive results of exit surveys, specifically whether the acceptability, feasibility, or perceived effectiveness of the program varied based on study group, and (2) descriptive results of exit surveys and interviews comparing intervention components and health coach preferences.

Concurrently with exit survey analyses, we also analyzed the content of exit interviews using AI-driven NLP and researcher-coded qualitative analysis. We used NLP first to characterize exit interviews, which included (1) topic modeling analysis with Latent Dirichlet Allocation (LDA; [32]) and (2) sentiment analysis with the Finn Årup Nielsen (AFINN) lexicon [33]. With a given number of topics, LDA determines the most likely topics within each document and the most likely terms within each topic [32]. The number of topics (*k*) used in our LDA was determined through a preliminary analysis using 3 methods [34]. Overall topic prevalence, or the topic’s proportion of a given document on average, is characterized by *Ɵ_k_*. Following the LDA, 2 study team members (FJG and OKE) named each topic based on recursive reading of interviews most likely to include each topic. These coders compared the names to ensure trustworthiness. For the sentiment analysis, we used the AFINN lexicon, which assigns values ranging from −3 to 3 based on their negative to positive valence, with 0 indicating neutrality in words. Documents are each given an index score based on the net value of included terms from the AFINN lexicon [33].

Based on the scope of the NLP results, study team members then conducted a rapid qualitative analysis on specific exit interview questions to target remaining areas of research interest. In total, 7 study team members (FJG, OKE, SK, MF, LL, and Holly Boyle and Sophia Sniffin) participated in the deductive qualitative coding process informed by the rigorous and accelerated data reduction (RADaR) technique for rapid qualitative analysis [35]. This technique involves a series of spreadsheets and data tables in which qualitative passages are successively reduced to derive themes [35]. A randomly selected subset (10/50, 20%) of interviews was independently coded by multiple coders to assess interrater reliability. Prereconciliation meeting Cohen kappa values between coding pairs ranged from 0.72 to 0.82, and postreconciliation meeting kappa values ranged from 0.90 to 0.97. The coding framework was revised based on reconciliation discussions, and remaining interviews were divided among coders who engaged in ongoing consultation and discussion to reduce coder drift and maintain trustworthiness. The researcher-coded qualitative findings from exit interviews were compared with quantitative NLP results from exit interviews using joint display methods, specifically an integrated results matrix [36]. For interview thematic results, we distinguish between primary aim results, which focus on intervention acceptability, feasibility, and perceived effectiveness, and exploratory aim results, which concentrated on comparisons between trial groups or intervention components and young adults’ health coaching preferences.

### Ethical Considerations

This research was approved by the Yale University institutional review board (2000030417). All eligible participants discussed an informed consent form in detail with a study team member before agreeing to take part in the study, and informed consent was obtained from every participant. During the informed consent process, the intervention’s focus on alcohol use and related physiological metrics was made explicit. Participants’ identifying information was kept private and confidential and stored only on a secure university server. All data used for the user experience evaluation were deidentified before analysis. Participants could earn up to US $279 if they completed all study activities (ie, smartphone diaries, study visits, and wearing and returning the Oura Ring).

## Results

### Sample

**Figure 1.**
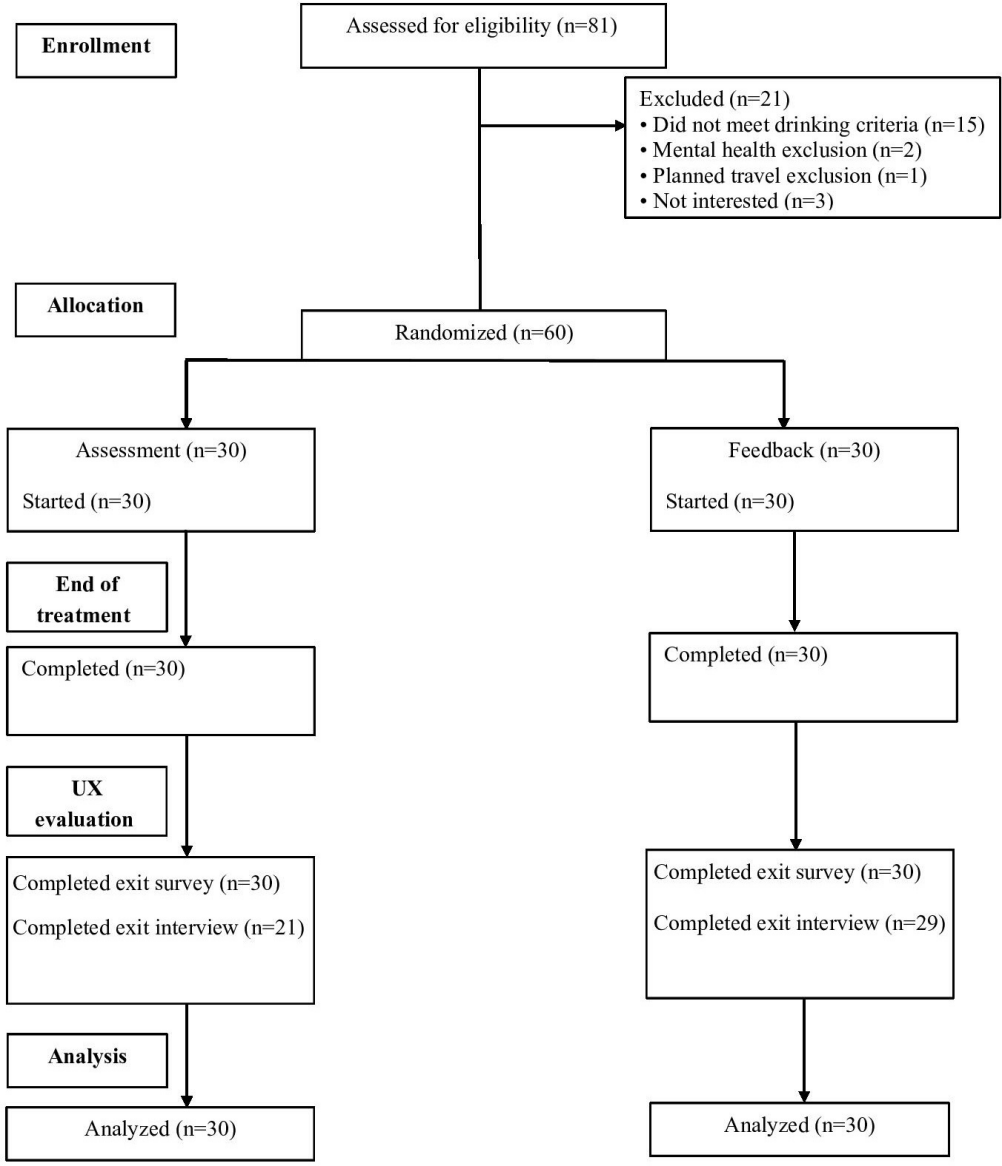
CONSORT (Consolidated Standards of Reporting Trials) flow diagram.

A total of 60 participants took part in the RCT and completed the exit survey (Figure 1). Almost all feedback group participants (n=29) and most assessment group participants (n=21) completed the exit interview. Half of the participants (30/60, 50%) were men, and 81.6% (49/60) were White, with a mean age of 22.02 (SD 1.98) years (Table 1). The majority (40/60, 66.6%) were students, and most (41/60, 68.3%) were employed. Over three-fourths (47/60, 78.3%) met criteria for an AUD, 21.7% (13/60) for another substance use disorder, and 15% (9/60) for an MHD. No demographic variable differed significantly between the assessment and feedback group participants.

**Table 1.** Sample characteristics (N=60).

Sample characteristic	Assessment (n=30), n (%)	Feedback (n=30), n (%)	Total (n=60)^a^, n (%)
Gender^b^			
Man	16 (53.3)	14 (46.6)	30 (50)
Woman	14 (46.7)	15 (50)	29 (48.3)
Nonbinary	0 (0)	1 (3.3)	1 (1.6)
Race			
Asian	2 (6.6)	1 (3.3)	3 (5)
Black	3 (10)	3 (10)	6 (10)
Multiracial	0 (0)	1 (3.3)	1 (1.6)
Other	1 (3.3)	0 (0)	1(1.6)
White	24 (80)	25 (83.3)	49 (81.6)
Ethnicity			
Not Hispanic or Latine	25 (83.3)	26 (86.6)	51 (85)
Hispanic or Latine	5 (16.6)	4 (13.3)	9 (15)
Student status			
Student	19 (63.3)	21 (70)	40 (66.6)
Nonstudent	11 (36.6)	9 (30)	20 (33.3)
Employment status			
Part-time	8 (26.7)	16 (53.3)	24 (40)
Not working	12 (40)	7 (23.3)	19 (31.7)
Full-time	10 (33.3)	7 (23.3)	17 (28.3)
Met criteria for AUD^c^ ^d^			
No	7 (23.3)	8 (26.7)	15 (25)
Mild	14 (46.7)	9 (30)	25 (41.7)
Moderate	7 (23.3)	9 (30)	16 (26.7)
Severe	2 (6.7)	4 (13.3)	6 (10)
Met criteria for other SUD^e^			
No	24 (80)	23 (76.7)	47 (78.3)
Yes	6 (20)	7 (23.3)	13 (21.7)
Ever AUD or SUD treatment			
No	30 (100)	29 (96.6)	59 (98.3)
Yes	0 (0)	1 (3.3)	1 (1.6)
Met criteria for a MHD^f^			
No	25 (83.3)	26 (86.7)	51 (85)
Yes	5 (16.6)	4 (13.3)	9 (15)

a Age=mean 22.02, SD 1.98, range 18.03‐25.94 years.

b “Gender” refers to participants’ self-identified gender identity, not biological sex.

c AUD: alcohol use disorder.

d Baseline alcohol use (past 28 d): Total standard alcoholic drinks=mean 73.91, SD 36.89; range 28.50‐219.49 drinks. Alcohol grams/day=mean 36.96, SD 18.45; range 14.25‐109.75 grams/day.

e SUD: substance use disorder.

f MHD: mental health disorder.

### Exit Survey

On a 100-point scale, participants in both groups reported high acceptability (mean 84.17, SD 17.81) and perceived effectiveness (eg, promoting hope [mean 70.42, SD 25] and meeting program goals [mean 75.83, SD 21.57]) of the overall program (Table 2). Almost all participants (58/60, 96.7%) said that they would recommend the program to a family member. Although participants in both groups found the overall program to be highly feasible (eg, schedule duration [mean 77.92, SD 25.25] and schedule workability [mean 91.67, SD 15.03]), assessment group participants reported some higher aspects of overall program feasibility compared with feedback group participants, including schedule workability (mean difference=8.30, *P*=.03) and visit comfortability (mean difference=10.00, *P*=.007) of visits.

**Table 2.** Exit survey results: rated agreement on a scale (0-100; n=60).

	Mean (SD)^a^, (1‐100)	Rated positive (>50), n (%)	Number
Acceptability			
Feedback report sleep/cardio information was likeable	92.67 (13.25)	56 (96.6)	58
Were willing to wear Oura Ring another week	90.83 (17.20)	55 (91.7)	60
Were willing to wear Oura Ring in future	89.17 (18.62)	55 (91.7)	60
Feedback report alcohol or substance info was likeable	87.50 (15.55)	30 (93.8)	32
Were willing to use Oura Ring with app in future	86.25 (20.80)	53 (88.3)	60
Oura Ring was not embarrassing to wear	84.58 (17.28)	57 (95)	60
Overall program was satisfying	84.17 (17.81)	52 (86.7)	60
Graphics in feedback report were acceptable	83.19 (17.76)	50 (86.2)	58
The quality of feedback report info was acceptable	82.33 (16.89)	53 (91.4)	58
Feedback report was interesting	76.72 (18.65)	46 (79.3)	58
Feedback report was visually appealing	76.29 (22.17)	43 (74.1)	58
Oura Ring was likeable	76.25 (22.75)	44 (73.3)	60
Feedback report layout was acceptable	75.00 (24.33)	43 (74.1)	58
Smartphone diary was likeable	59.17 (25.20)	26 (43.3)	60
Feasibility			
Oura Ring was not itchy	93.52 (13.77)	58 (96.7)	60
Oura Ring did not interfere with concentration	92.08 (14.18)	59 (98.3)	60
Overall program visits did not interfere with schedule	91.67 (15.03)	58 (96.7)	60
Oura Ring did not interfere with sleep	91.25 (16.48)	58 (96.7)	60
Oura Ring did not irritate skin	90.93 (15.38)	59 (98.3)	60
Overall program visits were comfortable	90.00 (14.70)	57 (95)	60
Oura Ring stayed on finger	87.92 (19.79)	55 (91.7)	60
Oura Ring did not interfere with activities	86.25 (18.65)	55 (91.7)	60
Oura Ring did not result in sweatiness	86.11 (18.14)	57 (95)	60
Feedback report alcohol/substance info was understandable	84.38 (18.78)	27 (84.4)	32
Smartphone diary was easy to complete	82.50 (20.74)	52 (86.7)	60
Oura Ring did not interfere with accessories	81.67 (24.30)	48 (80)	60
Oura Ring did not interfere with schedule	81.25 (28.61)	51 (85)	60
Smartphone diary did not interfere with schedule	80.83 (26.98)	49 (81.7)	60
The quantity of feedback report info was right	80.17 (17.99)	48 (82.8)	58
Remembered to wear and charge the Oura Ring	79.17 (23.55)	49 (81.7)	60
Feedback report sleep/cardio info was understandable	78.88 (21.87)	44 (75.9)	58
Overall program visits were not too long	77.92 (25.25)	46 (76.7)	60
Able to forget Oura Ring while wearing	76.67 (26.39)	47 (78.3)	60
Feedback report was understandable	70.26 (20.12)	37 (63.8)	58
Oura Ring did not interfere with exercise	70.00 (33.13)	39 (65)	60
Oura Ring was comfortable	68.33 (29.06)	42 (70)	60
Remembered to complete smartphone diary	65.42 (28.03)	39 (65)	60
Were willing to complete smartphone diary in an app	64.17 (28.51)	33 (55)	60
Smartphone diary was not burdensome	60.00 (27.69)	29 (48.3)	60
Perceived effectiveness			
Feedback report sleep/cardio information was helpful	88.36 (17.66)	53 (91.4)	58
Feedback report sleep tips were helpful	85.00 (18.10)	26 (86.7)	30
Feedback report alcohol/substance info was helpful	84.48 (18.03)	50 (86.2)	58
Feedback report alcohol use tips were helpful	81.90 (21.02)	22 (75.9)	29
Feedback report exercise tips were helpful	76.61 (21.35)	21 (67.7)	31
Overall program is effective in meeting its goals^b^	75.83 (21.57)	49 (81.7)	60
Feedback report stress-related tips were helpful	73.28 (28.29)	21 (72.4)	29
Feedback report diet tips were helpful	72.50 (25.72)	20 (66.7)	30
Overall program supports lifestyle goals	70.83 (20.15)	37 (61.7)	60
Overall program promotes hope	70.42 (25.00)	35 (58.3)	60
Feedback report substance use tips were helpful	69.64 (24.87)	16 (57.1)	28
Oura Ring app readiness tips were helpful	67.00 (29.51)	14 (56)	25
Oura Ring app bedtime tips were helpful	56.25 (34.77)	11 (45.8)	24
Oura Ring app activity prompts were helpful	52.88 (30.27)	9 (34.6)	26
Other			
Habits targeted by overall program were important	82.08 (20.11)	49 (81.7)	60
Oura Ring app use frequency (weekly to multiple daily)	83.33 (21.71)	28 (93.3)	30
Were willing to purchase Oura Ring	49.35 (29.10)	23 (45.1)	51
Were willing to purchase feedback report tips	43.96 (26.01)	19 (35.8)	53

a Mean (SD) are based on participants’ ratings of agreement with each statement on acceptability, feasibility, or perceived effectiveness on a 0‐100 scale. All values over 50 correspond to agreement with the statement (positive ratings of intervention features), and values over 75 indicate strong agreement. Not all participants were asked each question. Only feedback group participants answered questions about the Oura Ring app, and some questions were added later in the study and only answered by some participants. Calculations are based on the subset of participants who were asked each question, and no imputation methods were used with missing data. “Yes/No” exit survey items (eg, whether participants read all feedback reports) are reported in text.

b 83.3% (50/60) of participants described the overall program’s goals in part as learning about alcohol, alcohol’s relationship to sleep, and/or developing healthier drinking habits.

Related to their experiences of wearing the Oura Ring, participants in both groups reported high acceptability (eg, likeability [mean 76.25, SD 22.75] and willingness to continue wearing [mean 90.83, SD 17.20]) and feasibility (eg, ring comfortability [mean 68.33, SD 29.06] and no itchiness [mean 93.52, SD 13.77]). Most participants (44/51, 86.3%) said they would recommend the Oura Ring to a family member, and only 33.3% (20/60) noted marks on their skin from wearing it. Feedback group participants with full access to the Oura Ring mobile app also reported high acceptability (27/30, 90% liked the app) and moderate effectiveness (eg, activity prompt helpfulness [mean 52.88, SD 30.27] and readiness tip helpfulness [mean 67.00, SD 29.51]) of in-app recommendations and prompts.

Regarding the smartphone diaries, participants in both groups also reported moderate acceptability (mean 59.17, SD 25.20) and moderately high feasibility (eg, willingness to continue diaries in an app [mean 64.17, SD 28.51] and easiness [mean 82.50, SD 20.74]). Although diaries were highly rated in general, a descriptive comparison of ratings indicates that smartphone diaries may have been less acceptable and feasible than other intervention components (Table 2). Furthermore, members of both groups rated information in the feedback report as highly acceptable (eg, layout acceptability [mean 75.00, SD 24.33] and sleep or cardio information likeability [mean 92.67, SD 13.25]), feasible (eg, overall understandability [mean 70.26, SD 20.12] and alcohol or substance use information understandability [mean 84.38, SD 18.78]), and effective (eg, substance use tip helpfulness [mean 69.64, SD 24.87] and sleep or cardio information helpfulness [mean 88.36, SD 17.66]). A descriptive comparison of perceived effectiveness ratings (Table 2) indicates that the information in feedback reports may have been more effective than that in the Oura Ring app rated by feedback participants.

Feedback group participants had high self-reported adherence to intervention components, including using the app every day on average (mean 83.33, SD 21.71) on a 0‐100 scale from weekly to multiple daily use. The most frequently used aspects of the Oura Ring mobile app were sleep data (28/30, 93.3%), activity data (21/30, 70%), and cardiovascular recovery data (15/30, 50%). Less frequent in-app activities included tagging workouts (12/30, 40%), clicking on personal trend data (7/30, 23.3%), and interacting with story or meditation content (1/30, 3.3%). Most feedback group participants (24/30, 80%) also reported having read all 3 personalized feedback reports, and all feedback group participants (30/30, 100%) read at least 1 report. There was no significant difference between the baseline drinking levels of feedback group participants who read all 3 reports and those who did not (*P*=.35). Among all participants, they self-reported that they most frequently used sleep tips from the feedback reports (43/60, 71.7%), followed by alcohol or substance use tips (25/60, 41.7%), physical activity tips (19/60, 31.7%), stress management tips (18/60, 30%), and diet tips (14/60, 23.3%).

### Exit Interviews

#### NLP

**Figure 2.**
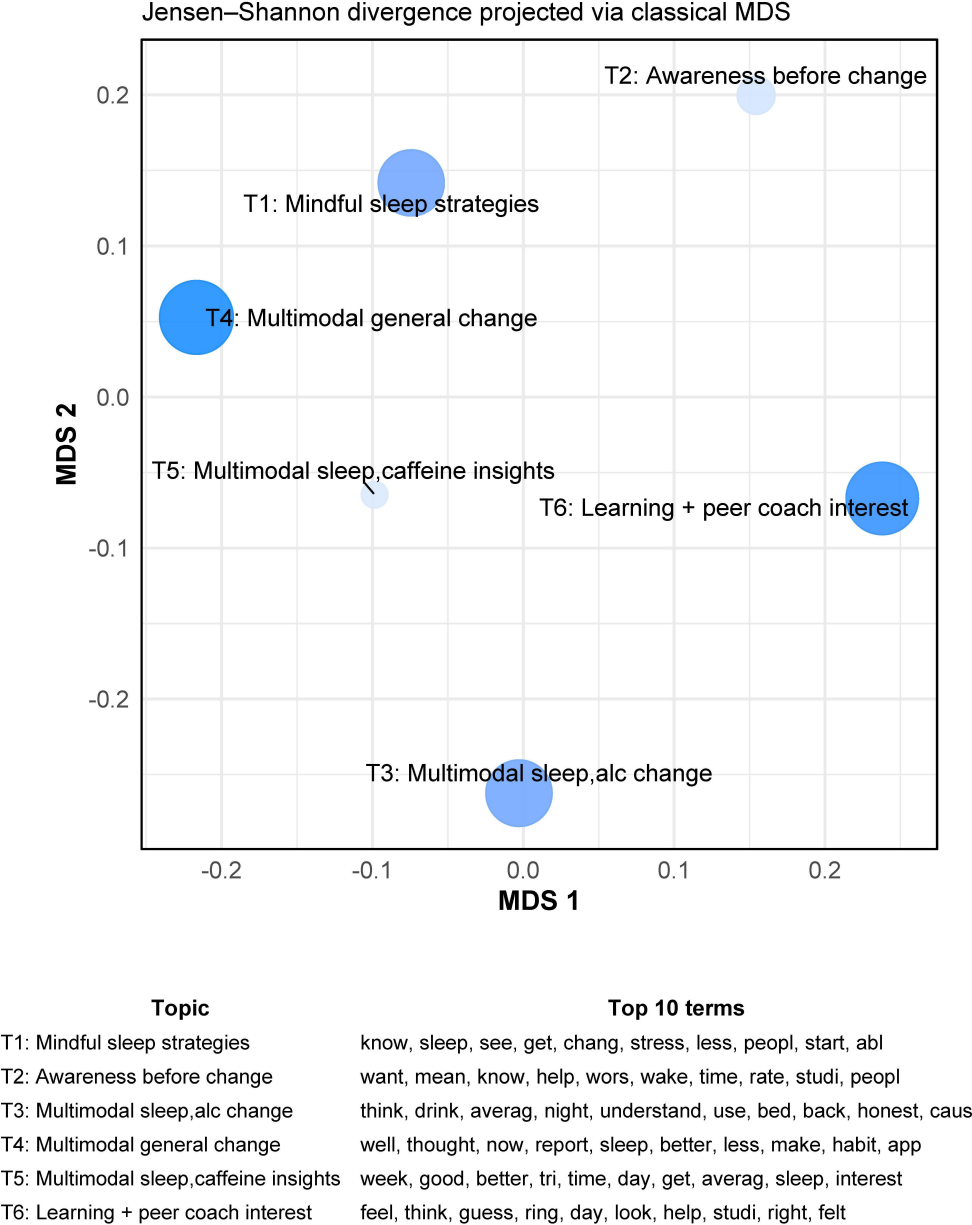
Feedback group exit interviews intertopic distance map (n=29). This map shows the distance between 6 topics within the Latent Dirichlet Allocation model based on pairwise Jensen–Shannon divergences between topic-word distributions. These were embedded in 2D using classical multidimensional scaling. Topics closer together on the map are more semantically similar. Larger point size and darker color indicate higher prevalence of a topic across exit interviews (*Ɵ_k_*). In order of prevalence, topic names based on recursive reading of interviews are: multimodal general change (*Ɵ_k_*=0.18); learning+peer coach interest (*Ɵ_k_*=0.18); multimodal sleep, alcohol change (*Ɵ_k_*=0.17); mindful sleep strategies (*Ɵ_k_*=0.17); awareness before change (*Ɵ_k_*=0.15); and multimodal sleep, caffeine insights (*Ɵ_k_*=0.15). The legend shows the 10 highest-probability terms within each topic. alc: alcohol; MDS: multidimensional scaling.

Among feedback exit interviews (n=29), 6 topics were modeled that were most likely to characterize each interview (Figure 2). The most common topic was multimodal general change (*Ɵ_k_=*0.18), in which “multimodal” refers to multiple helpful intervention components (Oura Ring, smartphone diaries, and feedback reports) that promoted general wellness in different domains, such as exercise, diet, and overall health and habits. Therefore, on average, 18% of each document was about this topic: general wellness changes related to multiple intervention components. The topic, learning+peer coach interest (*Ɵ_k_*=0.18)*,* or interest in peer coaching about the feedback report, was also prevalent. The next most common topics were multimodal sleep or alcohol change (*Ɵ_k_*=0.17), or multiple helpful program components specifically promoting sleep improvement and alcohol reduction, and mindful sleep strategies (*Ɵ_k_*=0.17), or trying mindfulness to improve sleep. These were followed by awareness before change (*Ɵ_k_*=0.15; or planning health changes based on personalized feedback reports) and multimodal sleep or caffeine insights (*Ɵ_k_*=0.15; or learning about sleep or caffeine from multiple helpful aspects of the program.

**Figure 3.**
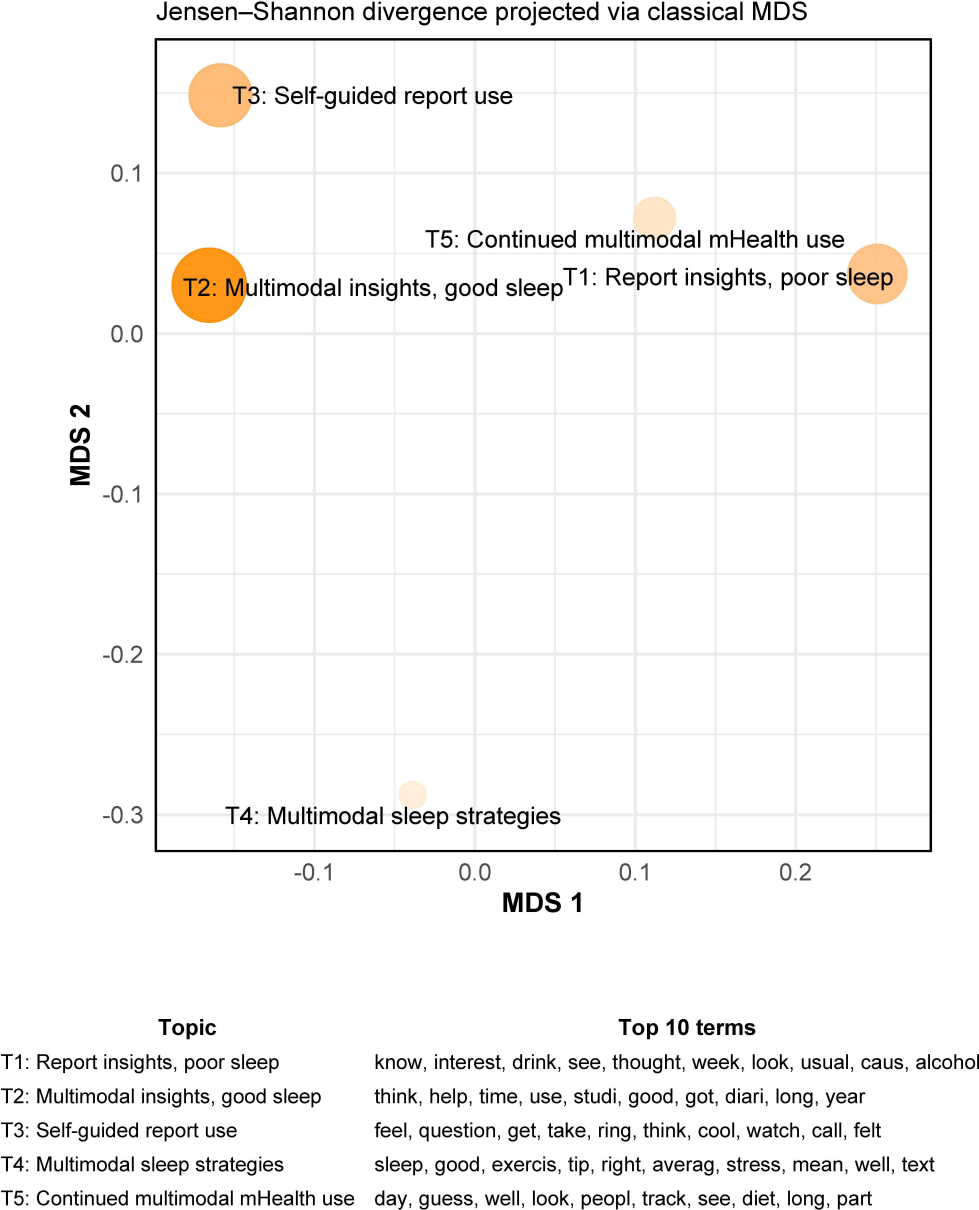
Assessment group exit interviews intertopic distance map (n=21). This map shows the distance between 5 topics within the Latent Dirichlet Allocation model based on pairwise Jensen–Shannon divergences between topic-word distributions. These were embedded in 2D using classical multidimensional scaling. Topics closer together on the map are more semantically similar. Larger point size and darker color indicate higher prevalence of a topic across exit interviews. In order of prevalence, topic names based on recursive reading of interviews are: multimodal insights, good sleep (*Ɵ_k_*=0.22), self-guided report use (*Ɵ_k_*=0.20); report insights, poor sleep (*Ɵ_k_*=0.20); continued multimodal mHealth use (*Ɵ_k_*=0.19); and multimodal sleep strategies (*Ɵ_k_*=0.18). MDS: multidimensional scaling; mHealth: mobile health.

We modeled 5 primary topics from the assessment exit interviews (n=21; Figure 3). The most common topic was multimodal insights, good sleep (*Ɵ_k_*=0.22), or learning about good sleep from multiple helpful components of the program. Next most common topics were self-guided report use (*Ɵ_k_*=0.20; or learning from the feedback report without a coach) and report insights, poor sleep (*Ɵ_k_*=0.20; or learning about sleep deficits from the feedback report). These were followed by continued multimodal mobile health use (*Ɵ_k_*=0.19), or finding multiple aspects of the program helpful due to previous use of mobile health, and multimodal sleep strategies (*Ɵ_k_*=0.18), or trying sleep strategies based on multiple helpful components of the program.

**Figure 4.**
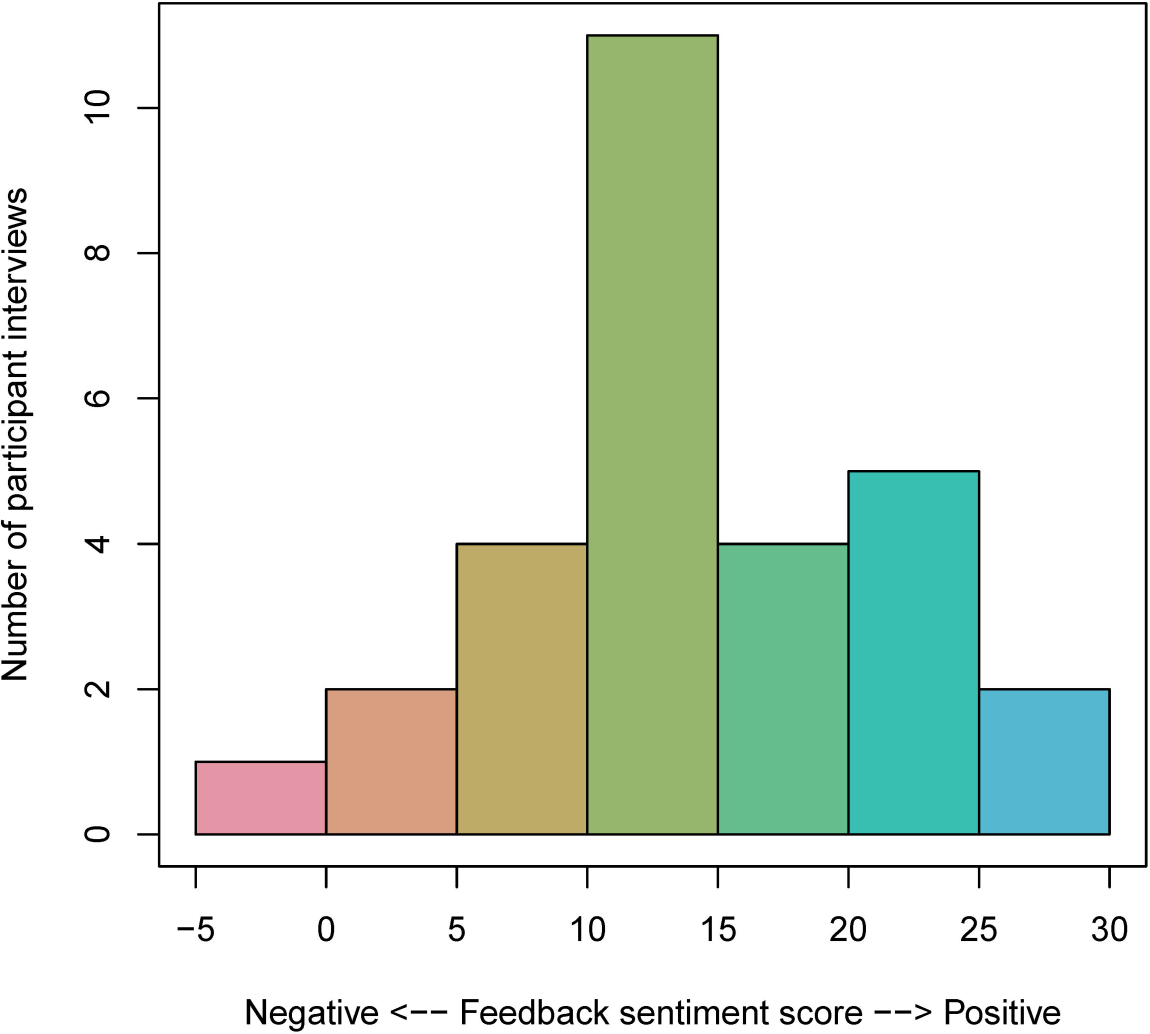
This histogram shows the frequency of sentiment scores (mean 14.66, SD 7.53; range −4 to 30) in the exit interviews of feedback group participants (n=29). These scores were calculated using the AFINN lexicon [33]. Positive sentiment scores indicate overall positively valenced words within an exit interview, whereas negative sentiment scores indicate negatively valenced words. Virtually all participants in the feedback group (28/29, 96.6%) had positive sentiment scores (>0), and 86.2% (25/29) had high positive sentiment (>10). AFINN: Finn Årup Nielsen.

Sentiment analysis showed generally positive perspectives among exit interviews in both the feedback group (mean 14.66, SD 7.53; range –4 to 30) and the assessment group (mean 15.57, SD 9.65; range 1‐32; Figures4  5). Positive sentiment scores indicate overall positively valenced words within an exit interview, whereas negative sentiment scores indicate negatively valenced words. Virtually, all participants in the feedback (28/29, 96.6%) and assessment (21/21, 100%) groups had positive sentiment scores (>0). However, on visual inspection, a larger proportion of feedback group participants (25/29, 86.2%) had high positive sentiment (>10) compared with the proportion of assessment group participants (15/21, 71.4%).

**Figure 5.**
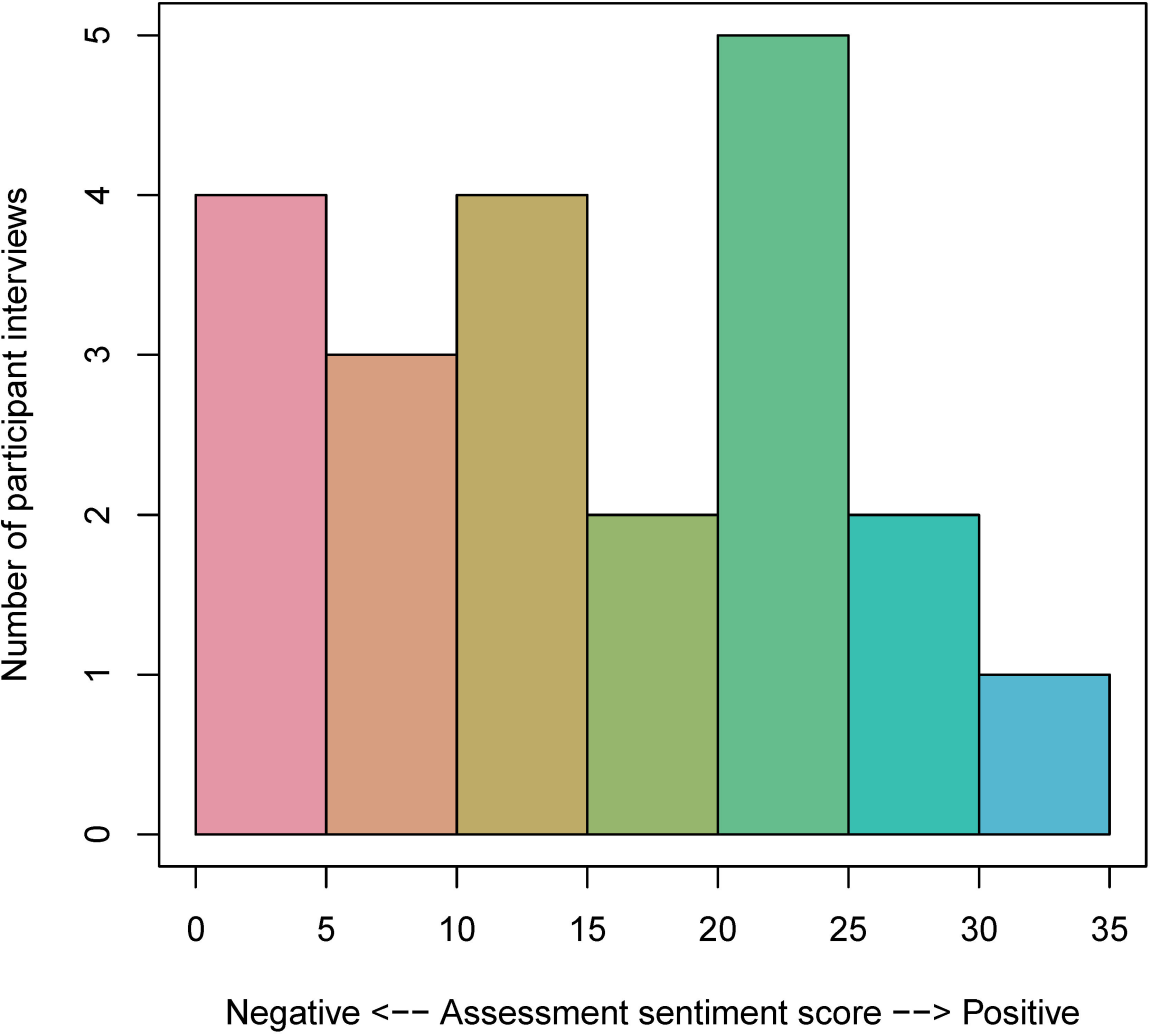
This histogram shows the frequency of sentiment scores (mean 15.57, SD 9.65; range 1‐32) in the exit interviews of assessment group participants (n=21). These scores were calculated using the AFINN lexicon [33]. Positive sentiment scores indicate overall positively valenced words within an exit interview, whereas negative sentiment scores indicate negatively valenced words. All assessment participants (21/21, 100%) had positive sentiment scores (>0), and 71.4% (15/21) had high positive sentiment (>10). AFINN: Finn Årup Nielsen.

#### Researcher-Coded Rapid Qualitative Analysis

Study team members conducted a targeted rapid qualitative analysis using the RADaR technique [35] (refer to table of themes, definitions, and salience in Multimedia Appendix 2). Based on exit interview questions with 50 participants across both groups, we identified 3 thematic categories: helpfulness and comparison of program components, report information and preferences, and program engagement and adherence. As detailed in the Multimedia Appendix 2, some questions were asked only of feedback group participants (eg, feedback report helpfulness), and some exploratory questions were added iteratively during the study (eg, health coaching preferences).

Furthermore, five themes within helpfulness and comparison of program components included (1) helpfulness of Oura Ring (asked of n=50 participants), (2) helpfulness of smartphone diaries (n=50), (3) helpfulness of feedback report (n=29 feedback group participants), (4) most influential: Oura, diaries, or report (exploratory result; n=24 feedback group participants), and (5) learned more: Oura versus report (exploratory result; n=16 feedback group participants). Most participants in both the feedback and assessment groups discussed helpful aspects of wearing the Oura Ring, and relatively small proportions of the feedback and assessment groups discussed unhelpful aspects or suggestions for improvement. One feedback group participant noted the helpfulness of wearing the Oura Ring and using the mobile app:

*I was able to see every morning...how much sleep I got...I was able to...make connections like, ‘Oh...I got six hours of sleep. No wonder why at 3:00 [PM], I’m...exhausted.*

Similarly, most participants in the feedback and assessment groups described helpful aspects of the smartphone diaries with comparatively small proportions of the feedback and assessment groups discussing unhelpful aspects of diaries or suggestions for improvement. One assessment group participant said:

*[The diary] was helpful. In some days, it helped me keep [my behaviors] in check.*

Only feedback group participants were asked about the helpfulness of feedback reports (asked of n=29 participants), and later in the study, as an exploratory question for a subset of participants, to compare program components (n=16‐24). Most found aspects of the feedback reports helpful, whereas very few found aspects of the reports unhelpful. One feedback group participant said of the report:

*One thing that was kind of crazy was...the amount of calories I drank [in alcohol]...that was helpful [information] because there was like 3500 calories essentially over the last two weeks.*

Similarly, another participant stated:

*The one factor [on the report] is...how many calories of alcohol someone drank...during the last two weeks. I think...if you’re not aware of that, that could be...a very helpful thing.*

Another stated:

*[The report] provided clarification too. It was just very...streamlined...in comparisons.*

When comparing different components, almost half of the feedback group participants who were asked this question stated that the Oura Ring was most influential, with about one-third preferring the feedback report, and one-fourth preferring smartphone diaries. One feedback group participant who selected the reports said:

*Probably the feedback [report], like the papers that you guys gave me, so that I was able to see...everything at once, rather than...just getting...a one-night thing from...the [Oura] Ring.*

Furthermore, the largest group of feedback group participants who were asked to compare what they learned stated that they knew more about their sleep from the Oura Ring app than the report, with one-fourth stating they learned equally from both. One feedback group participant who described learning from the Oura Ring said:

*Probably the Oura Ring, because I would look at...the ring every day. I see how I did [with sleep], so I feel like that was the most helpful.*

Themes in the report information and preferences category were based on questions added later in the study and asked of subsets of participants, including exploratory questions about health coach preferences. These five themes included (1) report: learned about sleep (asked of n=40 participants), (2) report: information amount (n=31), (3) report: health coach versus self-guided (n=32), (4) report: preferred coach type (n=30), and (5) report: preferred meeting mode (n=23). Among those who were asked what they learned about their sleep from the feedback report, the largest groups of feedback and assessment participants reported learning about their current sleep deficits, including the ways alcohol and other substances impacted their sleep. One feedback group participant reported:

*I learned about...the sleeping heart rate...being affected by alcohol...seeing how that’s an indicator of my sleep quality...even if I sleep for a long time, it doesn’t necessarily mean it’s good sleep.*

Another stated:

*I definitely...noticed like the heart rate and everything...drinking and before sleeping and while sleeping...just actually like realizing...what my BAC [Blood Alcohol Content] can get to...you don’t really think about that, you’re just like out having fun. So, I think you just made me...more mindful of my sleeping and drinking habits.*

Most participants who were asked about the amount of information in the report in the feedback and assessment groups thought sections had the right amount of information, whereas some who were asked thought some report sections had too much information. One assessment group participant who appreciated the amount of information in the report stated:

*I actually kind of like the lengthy list [of health tips] because you can like pick which [tip] works best for you...I think everything was really well explained and...split up into...different sections that made sense.*

As exploratory questions, participants in both groups were asked about their preferences for health coaching based on personalized data in their report. Among those asked, the largest group of participants in the feedback group was interested in health coaching, whereas the largest group among assessment participants preferred to read their report on their own. One feedback group participant said:

*I think meeting with someone would probably be better...to walk you through [the report], what it means and...I would be able to adjust and monitor weekly or biweekly.*

Regarding coach type, feedback group participants who were asked indicated a slight preference for a clinician over peer coaches, whereas assessment group participants had a slight preference for a peer coach over a clinician. One assessment participant said:

*I’m going to have to say [an] educated peer...only because I feel like some people can get the fear of doctors...and get overwhelmed by them.*

If they were to meet a health coach, most participants who were asked in the feedback and assessment groups preferred video teleconferencing (eg, Zoom [Zoom Communications]) or other remote methods compared with in-person health coaching.

Themes in the category of program engagement and adherence were based on questions added later in the study and asked of a subset of participants. The three themes were (1) considered dropping out of the program (asked of n=45), (2) motivation to participate in the program (n=44), and (3) motivation to stay engaged in the program (n=40). Almost all feedback and assessment group participants who were asked reported that they never considered dropping out of the program. Beyond financial compensation, the largest group of feedback and assessment participants who were asked reported that they joined the program due to curiosity about personalized health insights, to learn more about their wellness and connections to behaviors, like alcohol use. One assessment group participant said:

*I was interested on...my sleep and alcohol and how it’s affected.*

Similarly, another assessment group participant said:

*I...had questions...about my drinking and everything and wanted to see if it didn’t have...any impact on...everyday things like eating, sleeping and stress.*

A feedback group participant stated:

*I was curious about sleep for sure...I was very curious what this [Oura] Ring would do.*

As to what kept them engaged in the program, the largest group of feedback participants, almost half of those asked, cited SMS text reminders from study staff, and the largest group of assessment participants described that ease of use kept them engaged.

## Discussion

### Principal Results

Mixed methods evaluation results converged about users’ perceptions of the wearable physiological and behavioral feedback intervention (Table 3). Participants described the overall program as having high acceptability, feasibility, and perceived effectiveness in exit surveys and interviews. Wearing the Oura Ring was described as highly acceptable and feasible in the survey and as moderately to highly effective across both the survey and interview. These ratings included the Oura Ring mobile app for feedback group participants. Smartphone daily diaries tracking behavioral data were described as moderately to highly acceptable and feasible in the survey and as highly effective in the interview; therefore, the evaluation of this component had less cross-method convergence. The feedback reports were described as highly or moderately highly feasible and effective in both the exit survey and interviews and highly acceptable in the survey. Both methods used to analyze interviews (NLP and qualitative analysis) also showed that participants reported learning insights about their sleep deficits from the feedback reports.

**Table 3.** Convergent mixed methods results (asterisks denote findings that converged across methods).

Intervention	Exit survey	Exit interview	
		NLP^a^ (topics+sentiment)	Researcher-coded rapid qualitative analysis
Overall program	*High acceptability, feasibility, and effectiveness Assessment group feasibility > feedback group	*High acceptability *Effectiveness Feedback group effectiveness > assessment group	*High feasibility
Oura Ring or app	High acceptability and feasibility (Ring) *Moderate effectiveness (app)	—^b^	*High effectiveness Feedback group: most influential, learned more about sleep
Diaries	Moderate acceptability and moderate to high feasibility	—	High effectiveness
Feedback report	*High feasibility, effectiveness High acceptability	*Learning about sleep, especially deficits *Assessment group: prefer self-guided	*High feasibility, moderate to high effectiveness *Learning about sleep, especially deficits *Assessment group: prefer self-guided or peer-guided Feedback group: prefer coach, clinician All prefer remote coaching

a NLP: natural language processing.

b Not applicable.

Comparison of the feedback and assessment groups’ experiences revealed different findings depending on the method. Although the groups did not significantly differ on most exit survey ratings of the program, assessment group participants rated some aspects of program feasibility (comfortability and workability) more highly than feedback group participants. Also, per NLP with exit interviews, feedback group participants may have had higher perceived program effectiveness (reported behavior change).

Our exploratory analysis comparing program components revealed preferences for different aspects of program components. For example, some feedback group participants described the Oura Ring as most effective (influential on behavior) when asked in the exit interview. However, on their exit surveys, feedback group participants tended to rate the personalized information in the feedback reports as more effective (helpful) than the tips and prompts in the Oura Ring app. Also, although participants described the smartphone diaries as similarly effective (helpful) as other components (Oura Ring and feedback reports), they rated the acceptability and feasibility of the smartphone diaries as lower. These findings are consistent with previous research on lower levels of engagement in self-report data [37]. Actively completing the smartphone diaries (behavioral data) may have been more challenging than passively wearing the Oura Ring (physiological data). However, participants found the integration of both physiological and behavioral data streams in personalized feedback reports to be especially helpful.

Mixed methods analysis of exit interviews also revealed differences in participants’ preferences for health coaching about their personalized feedback. NLP and qualitative analysis of exit interviews indicated that assessment participants preferred to read their feedback reports independently without a health coach. The qualitative analysis revealed additional preferences, including feedback group participants’ interest in clinician health coaching and assessment group participants’ preference for peer coaching. The only discrepancy between evaluation methods was the topic highlighted within the NLP, which noted that feedback group participants are also interested in peer health coaching.

### Comparison With Previous Work and Future Directions

This evaluation paralleled previous research on young adults’ greater concern about improved wellness, such as improved sleep [6], rather than alcohol use. Study participants were motivated to join the study due to curiosity and interest in their wellness and personalized feedback, as opposed to a desire to reduce their drinking. Curiosity about highly personalized feedback also played a role in maintaining engagement in the intervention after its initiation. This aligns with previous findings that personalized feedback can enhance engagement [3]. Consistent with previous research, interventions focused on wellness goals may be more accessible and appealing to young adults than those primarily targeting alcohol use [5]. Despite their explicit focus on fitness and wellness, wearable devices have the potential to contribute to reducing risky behaviors.

Commercial wearable devices, like the Oura Ring, could incorporate more active monitoring of self-reported behaviors, such as alcohol use, to provide highly personalized feedback and foster motivational change in young adults. A key focus of our study was integrating behavioral self-monitoring and feedback, as this is not available in Oura. Although Oura users can make implicit associations by examining and tagging their physiological data, there is no explicit integrated feedback. Whereas it is especially challenging in general to encourage behavioral health app users to maintain engagement [38], our findings of high acceptability and feasibility support the integration of behavioral self-report data with passively collected physiological data. In particular, the combination of alcohol-related behavioral data and physiological data related to sleep and cardiovascular recovery could highlight connections between these data streams [23,25]. Our findings indicated that participants found the experience of active self-monitoring through smartphone diaries to be acceptable, feasible, and perceived as effective. Furthermore, they reported gaining insights from the integration of these data with their passively collected physiological data from the Oura Ring. Some noted they appreciated learning through integrated information and receiving tailored coaching. Insights into contextual factors that influence physiological data, such as sleep deficits, may promote dialogic reflection and enhance motivation to change risky behaviors [19,25].

Personalized feedback can be optimized to better promote insight and enhance change readiness. In this study, feedback group participants received daily health data and recent trends on the Oura Ring mobile app, along with more retrospective, integrated feedback in written reports every 2 weeks. Participants found both the feedback reports and the Oura Ring mobile app effective. On one hand, they reported preferences for the personalized insights in the integrated feedback reports; whereas, on the other hand, they liked the easy functionality of the Oura Ring and app.

Young adults may be interested in an option that combines the benefits of these intervention components (feedback reports and the Oura ring or app) via highly personalized, integrated in-app feedback. Mobile apps for wearable devices could offer active behavioral monitoring that is flexible according to the amount of time young adults are willing and able to answer self-report questions. Then, apps could offer integrated data reports at different time scales (eg, daily reports and longer trends) to leverage reflection-in-action and reflection-on-action [25]. Enhanced feedback options could also include opportunities for health coaching via educated peers or clinicians. Participants in both study groups showed some interest in peer coaching, and feedback group participants were more interested in clinician health coaching. Depending on the complexity of some data relationships (eg, HRV after a heavy drinking episode), it may be important to consult a coach to interpret and gain insights from personalized feedback reports.

Our results should be considered in the broader tradition of personalized feedback interventions for alcohol reduction. Normative feedback interventions compare young adults’ own drinking and perceptions of peer levels with actual peer levels, and these interventions may have small but meaningful impacts on alcohol reduction [21]. These interventions are theorized to address young adults’ social pressure to drink as a mechanism of change; however, highly personalized feedback on physiological and alcohol data may address overall wellness motivations to change. Given that young adults are generally unconcerned about their drinking [6], our findings reveal that the integration of personalized feedback on physiological metrics may increase the appeal of personalized alcohol feedback. Accessible, personalized feedback that promotes reflection [25] and engagement [3] may enhance young adults’ awareness of the connections between their behaviors and aspects of their wellness, like sleep and cardiovascular health. Further, integrated physiological and behavioral feedback has implications for other issues impacted by lifestyle behavior change, such as cardiovascular disease prevention.

### Limitations

Although our mixed methods user evaluation approach leveraged AI-driven approaches to enable breadth and depth [5], there were limitations in our methodology. Our sample size was relatively small for an RCT, especially for quantitative evaluation analyses. Furthermore, our sample consisted mostly of students from a single geographic location, which may not be representative of other young adults. Additionally, some survey and interview questions were introduced iteratively, limiting them to a subset of participants (eg, health coaching and component comparisons). The prevalence of these themes may have differed if they had been presented to the entire sample from the outset. Finally, as a phase 1 study primarily focused on feasibility, the duration of the intervention was only 6 weeks; however, a longer duration (>8 wk) would have been ideal to fully test the effect of an intervention intended to promote behavior change.

### Conclusions

Our results support the inclusion of self-report behavioral data in commercial wearable devices. Participants found the intervention acceptable, feasible, and effective, including the completion of smartphone diary self-monitoring. Many found that personalized feedback reports integrating their physiological and behavioral data were helpful and promoted insights about their sleep and other wellness goals. Wearable devices may lack important functionality by not capturing the behaviors that contribute to wellness goals, such as improved sleep, cardiovascular recovery, or fitness. Additionally, targeting risky and prevalent behaviors, such as alcohol use, through wearable devices could be a more appealing intervention for young adults who are less concerned about heavy drinking than about improving overall wellness.

## Supplementary material

10.2196/78613 Multimedia Appendix 1 CONSORT flow diagram.

10.2196/78613 Multimedia Appendix 2 Exit surveys, exit interview protocol, and exit interview themes.

10.2196/78613 Checklist 1 CONSORT-EHEALTH V1.6 checklist.

## Abbreviations

AFINN: Finn Årup Nielsen
AI: artificial intelligence
AUD: alcohol use disorder
CONSORT: Consolidated Standards of Reporting Trials
HRV: heart rate variability
MHD: mental health disorder
NLP: natural language processing
RCT: randomized controlled trial
