# Supplementary figures and images for: Oura Ring Behavioral Feedback Intervention for Alcohol Reduction in Young Adults: User Experience Evaluation of a Pilot Randomized Trial
Source: J Med Internet Res. 2025 Dec 4;27:e78613. doi: 10.2196/78613 (PMC12677873; doi:10.2196/78613)

### Supplementary Figure. CONSORT Flow Diagram

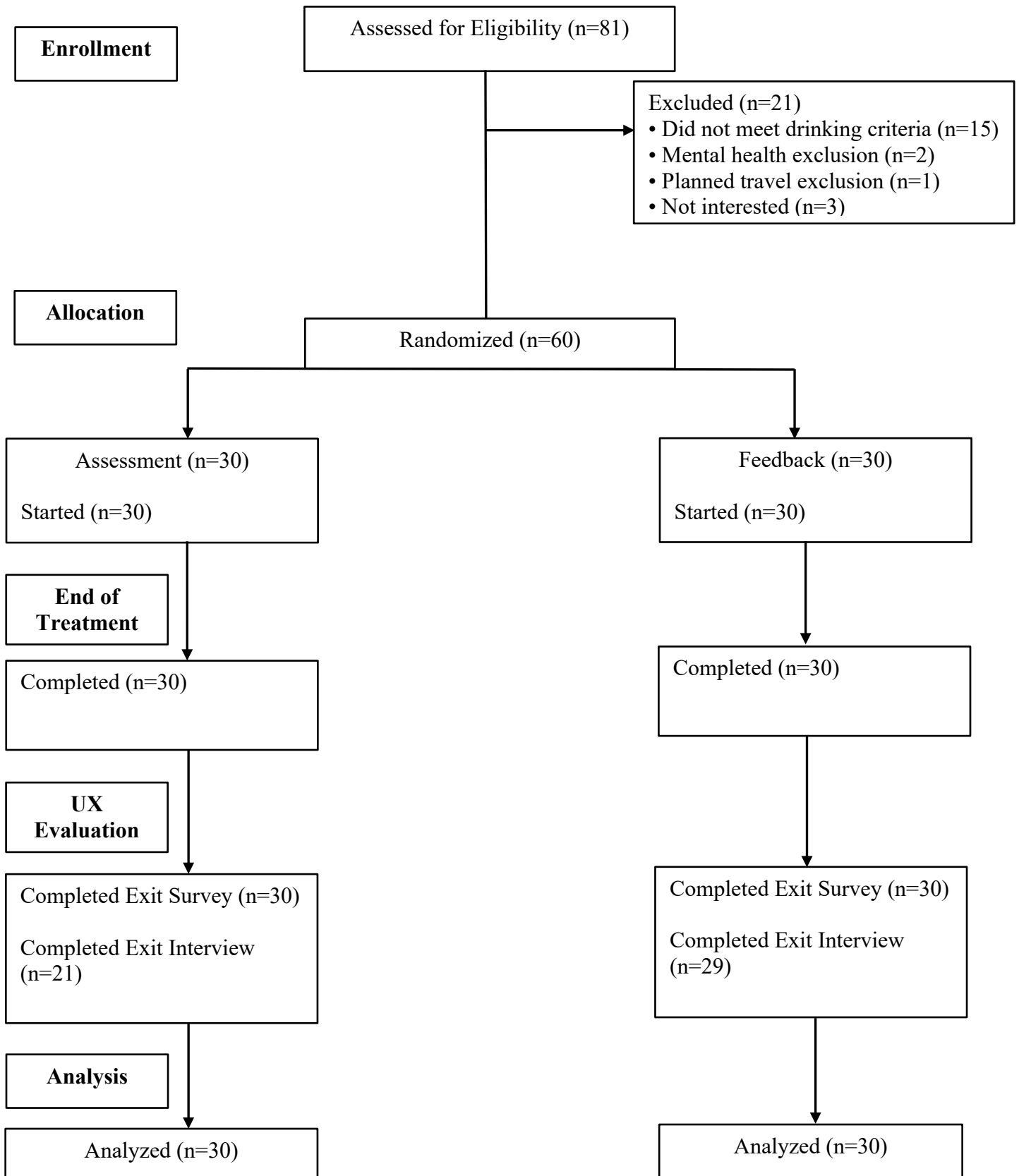

Supplement: Multimedia Appendix 1 [file jmir-v27-e78613-s001.pdf]
