# Supplementary material for: Oura Ring Behavioral Feedback Intervention for Alcohol Reduction in Young Adults: User Experience Evaluation of a Pilot Randomized Trial
Source: J Med Internet Res. 2025 Dec 4;27:e78613. doi: 10.2196/78613 (PMC12677873; doi:10.2196/78613)
Supplement: Multimedia Appendix 2 [file jmir-v27-e78613-s002.pdf]

Feedback Exit Survey

Please complete the survey below.

Thank you!

	Not at all satisfied		Neutral		Very satisfied
Overall, how satisfied were you with the study?	○	○	○	○	○

	Strongly disagree	Disagree	Neutral	Agree	Strongly agree
The study visits worked with my schedule.	○	○	○	○	○
The study visits were not too long.	○	○	○	○	○
Through the study, I felt that I could achieve healthy lifestyle goal changes.	○	○	○	○	○
I felt comfortable during the study visits.	○	○	○	○	○
Because of the study, I felt like there was a hope for change.	○	○	○	○	○
I believe the lifestyle habits targeted by this study are important.	○	○	○	○	○

What was it like to wear the OURA ring?

____

	Strongly Agree	Agree	Neutral	Disagree	Strongly Disagree
I liked wearing OURA.	○	○	○	○	○
OURA was physically uncomfortable.	○	○	○	○	○
Wearing OURA was embarrassing.	○	○	○	○	○
OURA interfered with my regular activities such as school, work, etc.	○	○	○	○	○
OURA fell off easily during my daily activities and/or sleep.	○	○	○	○	○
OURA interfered with my exercise.	○	○	○	○	○
OURA interfered with my sleep.	○	○	○	○	○
OURA interfered with my ability to concentrate.	○	○	○	○	○

OURA interfered with my choice of jewelry and accessories.	○	○	○	○	○
I found it difficult to remember to wear or charge OURA.	○	○	○	○	○
I often forgot I was even wearing OURA.	○	○	○	○	○
I did not have to change my daily routine (or give anything up) in order to comply with wearing OURA.	○	○	○	○	○
I would be willing to wear OURA for another week.	○	○	○	○	○
I would be willing to wear OURA again in the future.	○	○	○	○	○
If wearing OURA was part of a mobile health application, I would be willing to wear it and participate.	○	○	○	○	○

If you could change anything about the OURA ring, what would you change?

____

Did you notice any side effects from wearing OURA?

	1 (not noticeable)	2	3	4	5	6	7	8	9	10 (unbearable)
Itching	○	○	○	○	○	○	○	○	○	○
Sweating	○	○	○	○	○	○	○	○	○	○
Skin irritation	○	○	○	○	○	○	○	○	○	○

Did you have any marks on your skin from OURA?

○ Yes
○ No

What was it like to complete the daily diary entries?

____

	Strongly Agree	Agree	Neutral	Disagree	Strongly Disagree
I found it easy to complete my daily diary entry.	○	○	○	○	○
I found it burdensome to complete my daily diary entry.	○	○	○	○	○

I did not have to change my daily routine (or give anything up) in order to complete my diary entry.	○	○	○	○	○
I found it difficult to remember to fill out my daily diary entry.	○	○	○	○	○
I liked completing my daily diary entry.	○	○	○	○	○
If the daily diaries were part of a mobile health application, I would continue to complete them going forward.	○	○	○	○	○

If you could change anything about the diaries, what would you change?

____

Did you receive feedback on your sleep and health during weeks 2, 4, and 6?

○ Yes
○ No

Did you read all of the feedback reports (3 in total)?

☐ Yes I read all of the reports.
☐ I read two of the three reports.
☐ I read one of the three reports.
☐ I did not read any of the reports.

Why did you not read one or more of the reports?

____

	Did not like at all		Neutral		Greatly liked
How much did you like receiving information about your sleep and cardiovascular health?	○	○	○	○	○

	Not at all helpful		Neutral		Very helpful
How helpful was it to receive personalized information about your sleep and cardiovascular health?	○	○	○	○	○

	Not easy at all		Neutral		Very easy
How easy was it to understand the feedback on your sleep and cardiovascular health?	○	○	○	○	○

Is there anything you would change about the type of personalized sleep and cardiovascular health information received?

○ Yes ○ No

If yes, please explain.

____

	Did not like at all		Neutral		Greatly liked
How much did you like receiving information about your alcohol and other substance use?	○	○	○	○	○

	Not at all helpful		Neutral		Very helpful
How helpful was it to receive personalized information about your alcohol and other substance use?	○	○	○	○	○

	Not easy at all		Neutral		Very easy
How easy was it to understand the feedback on your alcohol and other substance use?	○	○	○	○	○

Is there anything you would change about the type of personalized alcohol use information you received? ○ Yes ○ No

If yes, please explain. __________________________________

Regardless of if you used the health tips provided in your feedback report, how helpful did you find the them?

	Not at all helpful		Neutral		Very helpful	Did not read these tips
Sleep Tips	○	○	○	○	○	○
Alcohol Tips	○	○	○	○	○	○
Substance Use Tips (Caffeine, Nicotine, Stimulant, etc.)	○	○	○	○	○	○
Physical Activity Tips	○	○	○	○	○	○
Stress Management Tips	○	○	○	○	○	○
Diet Tips	○	○	○	○	○	○

Which health tips did you follow from your feedback?

- ☐ Sleep tips
- ☐ Alcohol or substance use tips
- ☐ Physical activity tips
- ☐ Stress management tips
- ☐ Diet tips
- ☐ Did not follow tips

Think about your experience receiving your feedback reports at week 2, 4, and 6. Use the below scale to rate how good of job you believe the reports did.

	Inadequate	Poor	Acceptable	Good	Excellent
Interest	○	○	○	○	○
Visual appeal	○	○	○	○	○
Layout	○	○	○	○	○

Graphics	○	○	○	○	○
Ease of understanding information	○	○	○	○	○
Quality of information	○	○	○	○	○
Quantity of information	○	○	○	○	○

Did you like having access to the OURA mobile application?

○ Yes
○ No

Were there any specific OURA app features that you enjoyed or liked?

____

How often did you check the OURA app?

○ Multiple times a day
○ Daily
○ Several times a week
○ Weekly
○ Less than weekly
○ Did not check the app

What content did you interact with on the app? Check all that apply.

☐ Sleep data
☐ Recovery data
☐ Activity data
☐ Tagging workouts
☐ Clicking on your trends
☐ Story and meditation options
☐ Links to sleep, recovery, and other health information on the OURA blog
☐ Other
☐ I did not use the app

What else did you use on the app?

____

Did you follow any of these tips from OURA? If yes, rank how helpful you found the tip to be.

	Not at all helpful		Neutral		Very helpful	Did not use this tip
OURA's bedtime recommendations	○	○	○	○	○	○
OURA's activity prompts to move	○	○	○	○	○	○
OURA's readiness tips	○	○	○	○	○	○

Would you recommend this health promotion program to a friend or family member?

○ Yes ○ No

If no, please explain.

____

After reviewing your feedback report, how willing would you be to pay for a program that provides these personalized health data and tailored health tips on a regular basis?

Not Willing | Would Consider | Very Willing

(Place a mark on the scale above)

Would you recommend the OURA ring to a friend or family member?

- ◯ Yes
- ◯ No

After wearing the OURA ring, how willing would you be to purchase your own ring?

Not Willing | Would Consider | Very Willing

(Place a mark on the scale above)

What do you think was the purpose of this health promotion program?

____

	Not at all effective		Neutral		Very effective
How effective do you think the health promotion program was at achieving its purpose?	◯	◯	◯	◯	◯

Assessment Exit Survey

Please complete the survey below.

Thank you!

Did you receive feedback at week 10?

◯ Yes
◯ No

What were your first impressions upon receiving your feedback?

____

	Did not like at all		Neutral		Greatly liked
How much did you like receiving information about your sleep and cardiovascular health?	◯	◯	◯	◯	◯

	Not at all helpful		Neutral		Very helpful
How helpful was it to receive personalized information about your sleep and cardiovascular health?	◯	◯	◯	◯	◯

	Not easy at all		Neutral		Very easy
How easy was it to understand the feedback on your sleep and cardiovascular health?	◯	◯	◯	◯	◯

Is there anything you would change about the type of personalized sleep and cardiovascular health information received?

◯ Yes ◯ No

If yes, please explain.

____

	Did not like at all		Neutral		Greatly liked
How much did you like receiving information about your alcohol and other substance use?	◯	◯	◯	◯	◯

	Not at all helpful		Neutral		Very helpful
How helpful was it to receive personalized information about your alcohol and other substance use?	◯	◯	◯	◯	◯

	Not easy at all		Neutral		Very easy
How easy was it to understand the feedback on your alcohol and other substance use?	◯	◯	◯	◯	◯

Is there anything you would change about the type of personalized alcohol use information you received?

◯ Yes ◯ No

If yes, please explain.

____

How helpful did you find the tips from your feedback report?

	Not at all helpful		Neutral		Very helpful	Did not read these tips
Sleep Tips	◯	◯	◯	◯	◯	◯
Alcohol Tips	◯	◯	◯	◯	◯	◯
Substance Use Tips (Caffeine, Nicotine, Stimulant, etc.)	◯	◯	◯	◯	◯	◯
Physical Activity Tips	◯	◯	◯	◯	◯	◯
Stress Management Tips	◯	◯	◯	◯	◯	◯
Diet Tips	◯	◯	◯	◯	◯	◯

Which health tips do you intend to follow or try from your feedback?

- ☐ Sleep tips
- ☐ Alcohol or substance use tips
- ☐ Physical activity tips
- ☐ Stress management tips
- ☐ Diet tips
- ☐ Do not plan on trying tips

After reviewing your feedback report, how willing would you be to pay for a program that provides these personalized health data and tailored health tips on a regular basis?

Not Willing | Would Consider | Very Willing

(Place a mark on the scale above)

Think about your experience receiving your feedback report. Use the below scale to rate how good of job you believe the reports did.

	Inadequate	Poor	Acceptable	Good	Excellent
Interest	◯	◯	◯	◯	◯
Visual appeal	◯	◯	◯	◯	◯
Layout	◯	◯	◯	◯	◯
Graphics	◯	◯	◯	◯	◯
Ease of understanding information	◯	◯	◯	◯	◯
Quality of information	◯	◯	◯	◯	◯
Quantity of information	◯	◯	◯	◯	◯

Did you like having access to the OURA mobile application?

- ◯ Yes
- ◯ No

Were there any specific OURA app features that you enjoyed or liked?

____

What content did you interact with on the OURA app? Check all that apply.

- ☐ Tagging workouts
- ☐ Adding workouts
- ☐ Confirming naps
- ☐ Resource links/videos from OURA ("Why Sleep Matters" video, bedtime guidance link, etc.)
- ☐ Story and meditation options
- ☐ Ideal bedtime recommendation reminder
- ☐ Rest mode
- ☐ Other
- ☐ I did not use the app

	Not at all helpful		Neutral		Very helpful
How helpful was it to be able to tag workouts?	◯	◯	◯	◯	◯

How often did you tag workouts?

- ◯ Multiple times a day
- ◯ Daily
- ◯ Several times a week
- ◯ Weekly
- ◯ Less than weekly

	Not at all helpful		Neutral		Very helpful
How helpful was it to be able to add workouts?	◯	◯	◯	◯	◯

How often did you add workouts?

- ◯ Multiple times a day
- ◯ Daily
- ◯ Several times a week
- ◯ Weekly
- ◯ Less than weekly

	Not at all helpful		Neutral		Very helpful
How helpful was it to be able to confirm naps?	◯	◯	◯	◯	◯

How often did you confirm naps?

- ◯ Multiple times a day
- ◯ Daily
- ◯ Several times a week
- ◯ Weekly
- ◯ Less than weekly

	Not at all helpful		Neutral		Very helpful
How helpful was it to be able to view OURA's videos and resource links?	◯	◯	◯	◯	◯

How often did you view OURA's videos and resource links?

- ◯ Multiple times a day
- ◯ Daily
- ◯ Several times a week
- ◯ Weekly
- ◯ Less than weekly

	Not at all helpful		Neutral		Very helpful
How helpful was it to be able to use the story and meditation options?	◯	◯	◯	◯	◯

How often did you use the story and meditation options?

◯ Multiple times a day
◯ Daily
◯ Several times a week
◯ Weekly
◯ Less than weekly

	Not at all helpful		Neutral		Very helpful
How helpful was it to be able to receive ideal bedtime recommendation reminders?	◯	◯	◯	◯	◯

How often did you use the ideal bedtime recommendation reminders?

◯ Multiple times a day
◯ Daily
◯ Several times a week
◯ Weekly
◯ Less than weekly

	Not at all helpful		Neutral		Very helpful
How helpful was it to be able to use rest mode?	◯	◯	◯	◯	◯

How often did you use rest mode?

◯ Multiple times a day
◯ Daily
◯ Several times a week
◯ Weekly
◯ Less than weekly

What else did you use on the app?

____

How often did you check the OURA app?

◯ Multiple times a day
◯ Daily
◯ Several times a week
◯ Weekly
◯ Less than weekly
◯ Did not check the app

What did you use the OURA app for?

____

	Not at all helpful		Neutral		Very helpful	Did not use this tip
Did you find OURA's activity prompts to move helpful?	◯	◯	◯	◯	◯	◯

Oura Exit Interview: Feedback Group

1. What did you think about your sleeping habits before this program and what do you think now?
2. What did you think about your overall health before this program and what do you think now?
3. What did you think about your physical activity and diet/nutrition before this program and what do you think now?
4. What did you think about your alcohol consumption before this program and what do you think now? What about any other substances, if relevant?
5. What did you think about your stress level before this program and what do you think now?
6. Before starting this program, how did you think your behaviors compared to other people your age?
    a. Sleeping habits
    b. Overall health
    c. Physical activity
    d. Diet/nutrition
    e. Drinking habits
    f. Other substance use
    g. Stress level
7. Have you acquired any new health habits because of this study that you will continue going forward?
8. Tell us your reactions to the different part(s) of the study? Were any of them helpful/or not helpful (if any) and why:
    a. Knowing your habits were being monitored through biosensors
    b. Monitoring your habits yourself through daily diaries.
    c. Receiving personalized health feedback in report
9. Where were you able to learn more about your sleep, through the feedback reports we gave you or from the OURA app?
    a. And which feedback method did you prefer (paper vs in app)?
10. What did you think about the formatting of the report? What did you think about the amount of info given? What did you think about the type of info given? Did you think the report was overwhelming?
11. Did you read all three reports? If not, what got in the way?
12. Could you tell me something you learned specifically from the feedback report regarding...
    a. Your Sleep (page 1)
    b. Your SHRV/SHR- Check their understanding, and further explain if needed (page 2)
    c. Did you see a difference between your highest and lowest charts? Or notice anything about your alcohol use. (page 3)
    d. What tip did you learn? (page 4)
13. What do you think about...
    a. Having brief health coaching/ ability to walk through the report with someone?

    b. Or were you comfortable reviewing the info on your own?
    c. If yes to health coaching, telehealth, phone call in-person, text messaging?
    d. Would you rather the person be a coach (more motivational), Dr, or peer?
14. Would have wanted to be able to participate with other people also in the program? (opportunity to discuss experiences/ share your data with others?)
15. Having accomplishments better highlighted throughout the report?
16. Which of the above parts were most influential in changing your habits? [Oura app/device, diaries, or report]
17. If this program was not paid, do you think you would have still completed all necessary program components?
18. What drew you to this program? What did you like or dislike about the advertisement?
19. What helped to keep you participating once it started? Were there times when you thought of dropping out of the program?
20. If we asked you to participate longer what would encourage you to keep going?
21. Have you used other mhealth programs before? How long have/did you use them for?
22. What other habits would you have been interested to receive feedback about…
    a. That our devices and diaries measure (light, substance use, others?)
    b. that would require a new device or diary question to measure

Oura Exit Interview: Assessment Group

1. Tell us your reactions to the different parts of the study. Were any of them helpful/ or not helpful (if any) and why?
    a. Knowing your habits were being monitored through the biosensor
    b. Monitoring your habits yourself through daily diaries
2. Relating to your feedback report…
    a. Tell me something you learned about your sleep.
    b. Tell me what HR or HRV are/ what did you learn about yours?
    c. Tell me any tips you learned or which you want to try incorporating into your life
3. How did you like the tips? Is a long list helpful, or would it be better for you to pick and choose the topics you would like tips on?
4. Did you feel comfortable reviewing the information on your own in the feedback reports?
    a. Would it be beneficial to have a health coach to go through the report with you/
    b. would you set aside the time to meet with a coach? (telehealth, in-person, text message, none).
    c. Would you want the person to be more of a motivational coach, peer, or Dr?
5. Would you have wanted to have a connection with other people participating, having a group where you could discuss data and experiences?
6. Overall was the report overwhelming? Was there enough information or too much info? Was all of the content easy to understand? What information specifically was helpful or not helpful?
7. If this program was not paid, do you think you would have still completed all necessary program components?
8. What drew you to this program? What did you like or dislike about the advertisement?
9. What helped to keep you participating once it started? Were there times when you thought of dropping out of the program?
10. If this program was longer, is there any incentives that you think would of have helped you keep participating?
11. Have you ever used other mhealth programs? How long have you used?
12. What other habits would you have been interested to receive feedback about…
    a. That our devices and diaries measure (light, substance use, etc.)
    b. That would require a new device or diary question to measure

Supplementary Table. Exit interview themes from qualitative analysis

	Definition	*Assessment*	*Feedback*	Total [a]
Theme		*n* (% of 21)	*n* (% of 29)	*n* (% of 50)
Helpfulness and Comparison of Program Components (Both Groups)				
**Primary Result – Helpfulness of Oura Ring** (*n* = 50; 21 *Assessment*, 29 *Feedback*)				
Helpful	Aspects of the device or app promoted change, increased awareness, or captured interest.	17 (81%)	24 (82.8%)	41 (82.0%)
Unhelpful	Aspects of the device or app did not promote change, provide insight, or interest the participant.	2 (9.5%)	8 (27.6%)	10 (20%)
Neither	Aspects of the device or app were viewed neutrally, were not perceived as helpful or unhelpful.	3 (14.3%)	2 (6.9%)	6 (10%)
**Primary Result – Helpfulness of Smartphone Diaries** (*n* = 50; 21 *Assmt*, 29 *Feedback*)				
Theme	Definition	*Assessment*	*Feedback*	Total
Helpful	Completing the diaries promoted change, increased awareness, or captured interest.	19 (90.5%)	23 (79.3%)	42 (84%)
Unhelpful	Completing the diaries did not promote change, provide insight, or interest the participant.	3 (14.3%)	9 (31%)	12 (24%)
Neither	Completing the diaries was not perceived as helpful or unhelpful	1 (4.8%)	2 (6.9%)	3 (6%)
Report Information and Preferences				
**Primary Result – Report: Learned About Sleep** (*n* = 40; 19 *Assessment*, 21 *Feedback*)				
Theme	Definition	*Assessment*	*Feedback*	Total
Learned about sleep deficits	The report showed the participant how they had low sleep duration, efficiency, or another negative metric, including compared to their expectations or peers.	12 (63.2%)	15 (71.4%)	27 (67.5%)
Learned about sleep positives	The report showed the participant how they had high sleep duration, efficiency, or another positive	6 (31.6%)	3 (14.3%)	9 (22.5%)

	metric, including compared to their expectations or peers.			
Learned about sleep/ substance connections	The report helped the participant learn about the connection between sleep quality and substance use through personalized feedback.	1 (5.3%)	3 (14.3%)	4 (10%)
Learned nothing	The participant did not learn anything new about their sleep.	1 (5.3%)	2 (9.5%)	3 (7.5%)
**Primary Result – Report: Information Amount** (*n* = 31; 17 *Assessment*, 14 *Feedback*)				
Theme	Definition	*Assessment*	*Feedback*	Total
Right amount of information	The report had the right amount of information or was understandable in the format provided.	15 (88.2%)	11 (78.6%)	26 (83.9%)
Too much information	The report was overwhelming or difficult to understand due to the large amount of information in the provided format.	3 (17.6%)	6 (42.9%)	9 (29.0%)
**Exploratory Result – Report: Coach vs. Self-guided** (*n* = 32; 17 *Assmt*, 15 *Feedback*)				
Theme	Definition	*Assessment*	*Feedback*	Total
Self-Guided	The participant expressed interest in reviewing feedback reports themselves as opposed to going over them with a coach.	13 (76.5%)	4 (26.7%)	17 (53.1%)
Coach	The participant expressed interest in a health coach going over their report with them to provide personalized coaching and tailored health tips.	5 (29.4%)	10 (66.7%)	15 (46.9%)
**Exploratory Result – Report: Preferred Coach Type** (*n* = 30; 16 *Assessment*, 14 *Feedback*)				
Theme	Definition	*Assessment*	*Feedback*	Total
Peer	A peer coach or motivational coach without credentials (no license, not an MD, etc.) is generally preferred. This peer would be a facilitator with some training.	9 (56.3%)	4 (28.6%)	13 (43.3%)

Clinician	A clinician with credentials or doctor is generally preferred as a health coach.	7 (43.8%)	6 (42.9%)	13 (43.3%)
No preference	The participant does not have a preference between types of coaches or answered this question despite not wanting to speak to a coach.	2 (12.5%)	5 (35.7%)	7 (23.3%)
**Exploratory Result – Report: Preferred Meeting Mode** (*n* = 23; 10 *Assmt*, 13 *Feedback*)				
Theme	Definition	*Assessment*	*Feedback*	Total
Zoom	The participant noted they would like to meet with a health coach over Zoom or other video call.	7 (70%)	10 (76.9%)	17 (73.9%)
Phone	The participant noted they would like to meet with a health coach over the phone (audio call only).	2 (20%)	5 (38.5%)	7 (30.4%)
Text	The participant noted they would like to meet with a health coach using asynchronous text messaging or chatting.	0 (0%)	4 (30.8%)	4 (17.4%)
In-person	The participant noted they would like to meet with a health coach in-person.	1 (10%)	0 (0%)	1 (4.3%)
No preference	The participant indicated that they don't care how they meet with a health coach or they answered this question, but don't really want to meet with a health coach.	1 (10%)	1 (7.7%)	2 (8.7%)
Program Engagement and Adherence				
**Primary Result – Considered Dropping out of Program** (*n* = 45; 19 *Assmt*, 26 *Feedback*)				
Theme	Definition	*Assessment*	*Feedback*	Total
Never considered	The participant never considered dropping out of the study and continued to adhere to study activities.	18 (94.7%)	23 (88.5%)	41 (91.1%)
Considered	The participant considered dropping out of the study at some	1 (5.3%)	3 (11.5%)	4 (8.9%)

	point even if they changed their mind.			
**Primary Result – Motivation to Participate in Program** (*n* = 44; 18 *Assmt*, 26 *Feedback*)				
Theme	Definition	*Assessment*	*Feedback*	Total
Curious about health	The participant joined the program primarily to learn personalized health insights and about connections between wellness and behaviors.	8 (44.4%)	17 (65.4%)	26 (59.1%)
Financial compensation only	The participant solely joined the program due to monetary incentives.	6 (33.3%)	2 (7.7%)	9 (20.5%)
Enjoy research	The participant joined the program primarily because they enjoy taking part in research.	2 (11.1%)	2 (7.7%)	4 (9.1%)
Sleep challenges	The participant was motivated by problems with their sleeping to take part in the program.	1 (5.6%)	2 (7.7%)	3 (6.8%)
Alcohol challenges	The participant was motivated by problems with their alcohol use to take part in the program.	1 (5.6%)	1 (3.8%)	2 (4.5%)
Participation was easy	The participant took part in the program primarily because it seemed relatively easy to participate.	1 (5.6%)	1 (3.8%)	2 (4.5%)
**Primary Result – Motivation to Stay Engaged in Program** (*n* = 40; 15 *Assmt*, 25 *Feedback*)				
Theme	Definition	*Assessment*	*Feedback*	Total
Text reminders	Text messages from study staff were most helpful in keeping the participant engaged in the study.	3 (20%)	11 (44%)	14 (31.8%)
Curiosity about personal data	Wanting to know more about their personalized health information or data kept the participant engaged in the study.	5 (33.3%)	9 (36%)	14 (31.8%)
Ease of use	Easiness of taking part kept the participant engaged in the study.	6 (40%)	2 (8%)	8 (18.2%)

In-app reminders	Notifications through the Oura app/device were most helpful in keeping the participant engaged in the study.	1 (6.7%)	6 (24%)	7 (15.9%)
Habit	Study procedures becoming habitual kept the participant engaged in the study.	1 (6.7%)	3 (12%)	4 (9.1%)
Financial compensation	Study payments kept the participant engaged in the study.	1 (6.7%)	1 (4%)	2 (4.5%)
Nothing	Nothing in particular was most influential in keeping the participant engaged in the study	0 (0%)	2 (8%)	2 (4.5%)
Helpfulness and Comparison of Program Components (Feedback Group Only)				
**Primary Result – Helpfulness of Feedback Report** (*n* = 29 *Feedback*)				
Theme	Definition	*Assessment*	*Feedback*	Total
Helpful	Reading the report promoted change, increased awareness, or captured interest.	-	25 (86.2%)	25 (86.2%)
Unhelpful	Reading the report did not promote change, provide insight, or interest the participant.	-	3 (10.3%)	3 (10.3%)
Neither	Reading the report was not perceived as helpful or unhelpful, such that they did not think deeply about it or consider it new information.	-	3 (10.3%)	3 (10.3%)
**Exploratory Result – Most Influential: Oura, Diaries, or Report** (*n* = 24 *Feedback*)				
Theme	Definition	*Assessment*	*Feedback*	Total
Oura Ring	The app/device was most motivating for the participant to change their behavior.	-	10 (41.7%)	10 (41.7%)
Report	The report was most influential for participants to change their behavior.	-	7 (29.2%)	7 (29.2%)
Smartphone diaries	The diaries were most motivating for the participant to change their behavior.	-	6 (25%)	6 (25%)

**Exploratory Result – Learned More: Oura vs. Report** (*n* = 16 *Feedback*)				
Theme	Definition	*Assessment*	*Feedback*	Total
Oura Ring	The device or app taught the participant more useful information about their sleep, increased their awareness, or they generally preferred this format for learning.	-	8 (50%)	8 (50%)
Feedback report	The report taught the participant more useful information about their sleep, increased their awareness, or they generally preferred this format for learning.	-	3 (18.8%)	3 (18.8%)
Both	The participant equally described ways that both the app/device and the report helped them learn in different ways about their sleep.	-	4 (25%)	4 (25%)

Note: Proportions are based on the responses of participants asked each question, not all participants were asked each question because some questions were added iteratively later in the study.
